# From Bacterial Extract to Breakthrough Therapy: *Pseudomonas fluorescens*-Enabled Green Synthesis of pH-Responsive Chitosan–Silver Hybrid Nanoparticles for Next-Generation Pulmonary Drug Delivery Anti-MDR Treatment

**DOI:** 10.3390/pharmaceutics17121527

**Published:** 2025-11-27

**Authors:** Khulood Fahad Alabbosh, Alaa Elmetwalli, Naseh A. Algehainy, Faisal H. Altemani

**Affiliations:** 1 Department of Biology, College of Science, University of Hail, Hail 55473, Saudi Arabia; 2 Prince Fahad bin Sultan Chair for Biomedical Research, University of Tabuk, Tabuk 71491, Saudi Arabia; 3 Department of Medical Laboratory Technology, Faculty of Applied Medical Sciences, University of Tabuk, Tabuk 71491, Saudi Arabia

**Keywords:** green synthesis, chitosan-AgNPs, multidrug resistance, pulmonary delivery, biofilm disruption, antimicrobial nanomedicine, stimuli-responsive, inhalation therapy

## Abstract

**Background:** Multidrug-resistant (MDR) pulmonary infections represent a critical global health challenge, necessitating innovative therapeutic approaches. Green synthesis methodologies offer sustainable alternatives for nanoparticle fabrication while addressing antimicrobial resistance. **Methods:** Stimuli-responsive chitosan–silver hybrid nanoparticles (CS–Ag HNPs) were biosynthesized using *Pseudomonas fluorescens* bacterial extracts and loaded with ciprofloxacin for targeted pulmonary delivery. Comprehensive characterization included dynamic light scattering, transmission electron microscopy, UV–visible spectroscopy, and aerodynamic assessment via next-generation impactor. Antimicrobial efficacy was evaluated against MDR *Pseudomonas aeruginosa* and *Klebsiella pneumoniae*, including biofilm disruption studies, and biocompatibility was assessed. Molecular docking analysis elucidated binding mechanisms. Cytotoxicity and epithelial barrier integrity were evaluated using Calu-3 cell models. **Results:** The biosynthesized NPs exhibited optimal physicochemical properties (180 ± 20 nm, PDI 0.21 ± 0.04, ζ-potential + 32.4 ± 3.1 mV) with high encapsulation efficiency (68.2 ± 4.0%). Aerodynamic analysis revealed excellent inhalation characteristics (MMAD 2.6 μm, FPF 65 ± 5%). The hybrid system demonstrated 4-fold enhanced antimicrobial activity against MDR pathogens and significant biofilm disruption (70% for *P. aeruginosa*, 65% for *K. pneumoniae*) compared to free ciprofloxacin. Cell viability remained ≥85% at therapeutic concentrations. Molecular docking revealed enhanced drug-target binding affinity (−11.2 vs. −9.3 kcal/mol) and multi-residue interactions. **Conclusions:** Green-synthesized CS–Ag HNPs represent a promising sustainable platform for combating pulmonary MDR infections through enhanced antimicrobial efficacy and optimal aerodynamic properties.

## 1. Introduction

The emergence and proliferation of multidrug-resistant (MDR) pathogens represent one of the most pressing challenges in contemporary medicine, with pulmonary infections being particularly devastating due to their high morbidity and mortality rates [1,2]. The World Health Organization has identified antimicrobial resistance (AMR) as a top-ten global public health threat, with MDR tuberculosis, *Pseudomonas aeruginosa*, and *Klebsiella pneumoniae* classified as critical priority pathogens requiring urgent research and development of novel therapeutic interventions [3,4].

Pulmonary MDR infections present unique therapeutic challenges due to the anatomical and physiological barriers of the respiratory tract, including mucociliary clearance, alveolar macrophage uptake, and the formation of persistent biofilms that significantly reduce drug penetration and efficacy [5,6]. Current treatment regimens for MDR pulmonary infections often involve systemic administration of high-dose antibiotics, leading to severe adverse effects, poor patient compliance, and further selection pressure for resistance development [7,8]. The limited penetration of systemically administered drugs into infected lung tissues, combined with the protective biofilm matrix, necessitates innovative drug delivery approaches that can overcome these barriers while minimizing systemic toxicity [9,10].

Nanotechnology-based drug delivery systems have emerged as promising solutions for addressing the limitations of conventional antimicrobial therapy [11,12]. Among various nanocarrier systems, chitosan-based NPs have gained significant attention due to their biocompatibility, biodegradability, mucoadhesive properties, and intrinsic antimicrobial activity [13,14]. The incorporation of silver NPs (AgNPs) into chitosan (CS) matrices creates hybrid systems with synergistic antimicrobial effects, combining the broad-spectrum bactericidal properties of silver with the biofilm-disrupting capabilities of CS [15,16].

The concept of green synthesis has revolutionized nanoparticle fabrication by utilizing biological systems, including bacteria, fungi, and plant extracts, as eco-friendly alternatives to conventional chemical and physical methods [17,18]. Microbiology-driven synthesis approaches offer several advantages, including reduced environmental impact, elimination of toxic substances, enhanced biocompatibility, and the potential for scalable production [19,20]. Recent studies have demonstrated that bacterial extracts can serve as both reducing and stabilizing agents in nanoparticle synthesis, producing particles with unique surface properties and enhanced biological activity [21,22].

Pulmonary drug delivery via inhalation offers distinct advantages for treating respiratory infections, including direct drug deposition at the infection site, reduced systemic exposure, lower dose requirements, and improved patient compliance [23,24]. However, successful pulmonary delivery requires careful consideration of aerodynamic properties, with particles exhibiting a mass median aerodynamic diameter (MMAD) of 1–5 μm being optimal for deep lung penetration and alveolar deposition [25,26]. Additionally, stimuli-responsive systems that can respond to the pathological microenvironment, such as altered pH in infected tissues, offer opportunities for controlled and targeted drug release [27,28].

Despite significant advances in antimicrobial nanotechnology, there remains a critical gap in the development of sustainable, green-synthesized hybrid nanoparticle systems specifically designed for inhalable delivery against pulmonary MDR infections. The combination of environmentally conscious synthesis methodologies with advanced nanomedicine approaches represents an innovative strategy that addresses both therapeutic efficacy and environmental sustainability concerns [29,30].

The primary objective of this study was to develop and comprehensively evaluate green-synthesized, stimuli-responsive CS-Ag HNPs loaded with ciprofloxacin for targeted pulmonary delivery against MDR pathogens. Specifically, we aimed to: (1) establish a reproducible microbiology-based synthesis protocol using *Pseudomonas fluorescens* bacterial extracts; (2) optimize nanoparticle formulation parameters to achieve ideal physicochemical and aerodynamic properties for inhalation; (3) evaluate pH-responsive drug release behavior under simulated lung fluid conditions; (4) assess antimicrobial efficacy and biofilm disruption capabilities against clinically relevant MDR strains; and (5) determine biocompatibility and epithelial barrier integrity using relevant cell culture models.

## 2. Materials and Methods

### 2.1. Materials

CS (medium molecular weight, 75–85% deacetylated), silver nitrate (AgNO_3_), ciprofloxacin hydrochloride, tripolyphosphate (TPP), acetic acid, and phosphate-buffered saline (PBS) were purchased from Sigma-Aldrich (St. Louis, MO, USA). *Pseudomonas fluorescens* ATCC 13525 was obtained from the American Type Culture Collection, Manassas, VA, USA. Mueller-Hinton broth, tryptic soy broth, and microbiological media components were sourced from BD Difco (Franklin Lakes, NJ, USA). Calu-3 human airway epithelial cells were acquired from ATCC. Cell culture media, fetal bovine serum, and antibiotics were obtained from Gibco (Thermo Fisher Scientific, Waltham, MA, USA). All chemicals were of analytical grade and used without further purification. Deionized water (18.2 MΩ·cm) was used throughout all experiments.

### 2.2. Preparation of Bacterial Extract

*Pseudomonas fluorescens* ATCC 13525 was cultured in tryptic soy broth at 28 °C with continuous shaking (150 rpm) for 24 h. The bacterial culture was centrifuged at 8000 rpm for 10 min, and the cell pellet was washed three times with sterile deionized water. The washed biomass was resuspended in deionized water (1:10 *w*/*v* ratio) and subjected to sonication (40 kHz, 30 min) followed by boiling at 100 °C for 15 min to release intracellular components. The resulting bacterial extract was centrifuged at 10,000 rpm for 15 min, and the clear supernatant was collected, filter-sterilized (0.22 μm), and stored at 4 °C until use. Protein concentration in the extract was determined using the Bradford assay [31].

### 2.3. Green Synthesis of Chitosan–Silver Hybrid NPs

CS solution (0.5% *w*/*v*) was prepared by dissolving CS in 1% acetic acid with continuous stirring for 2 h at room temperature. The solution was filtered through a 0.45 μm membrane to remove undissolved particles. For silver nanoparticle synthesis, 10 mL of bacterial extract was mixed with 90 mL of 1 mM AgNO_3_ solution and incubated at 37 °C for 2 h under dark conditions. The formation of AgNPs was confirmed by the appearance of brownish coloration and UV–visible spectroscopy [31].

HNPs were prepared using the ionic gelation method [31]. Briefly, 20 mL of CS solution was mixed with 10 mL of biosynthesized AgNPs suspension under magnetic stirring (500 rpm). TPP solution (0.5% *w*/*v*) was added dropwise to achieve a chitosan: TPP ratio of 3:1 (*w*/*w*). The mixture was stirred for 30 min at room temperature, followed by sonication for 5 min to ensure homogeneous distribution. The resulting NPs were collected by centrifugation (15,000 rpm, 30 min), washed three times with deionized water, and lyophilized for storage [32].

For consistency, all bacterial extracts were prepared under standardized conditions. *Pseudomonas aeruginosa* (ATCC 27853) cultures were grown in nutrient broth (pH 7.2 ± 0.1) at 37 °C and 150 rpm for 18 h until OD_600_ = 0.8 ± 0.02. Cultures were centrifuged (10,000× *g*, 15 min), and the supernatant was filtered (0.22 µm). Each batch of extract was analyzed for pH and total soluble protein concentration (Bradford assay), which remained within ±5% across replicates. Three independent synthesis batches of CS–Ag HNPs were conducted using these extracts, and the physicochemical properties of the NPs were compared to confirm reproducibility.

### 2.4. Drug Loading

Ciprofloxacin-loaded HNPs were prepared by incorporating the drug during the ionic gelation process. Ciprofloxacin hydrochloride (10 mg) was dissolved in the chitosan–silver nanoparticle suspension before TPP addition. The drug-loaded NPs were collected and washed as described above. Encapsulation efficiency (EE%) and loading efficiency (LE%) were determined using high-performance liquid chromatography (HPLC) analysis of drug content in the NPs and the supernatant. Quantification of ciprofloxacin encapsulated within the NPs was performed using high-performance liquid chromatography (HPLC, Agilent 1260 Infinity II, Agilent Technologies, Santa Clara, CA, USA) equipped with a quaternary pump, autosampler, and UV–visible detector set at 278 nm. Separation was achieved on a reversed-phase C18 column (250 mm × 4.6 mm, 5 µm; Phenomenex, Torrance, CA, USA) maintained at 30 °C [33].

The mobile phase consisted of 0.1% (*v*/*v*) orthophosphoric acid in water (pH 3.0, adjusted with triethylamine) and acetonitrile in an 80:20 (*v*/*v*) ratio, delivered isocratically at a flow rate of 1.0 mL min^−1^. The injection volume was 20 µL, and the total run time was 10 min. Calibration curves were constructed from ciprofloxacin standard solutions in the concentration range 0.5–100 µg mL^−1^, showing excellent linearity (R^2^ = 0.999).

For sample preparation, an accurately weighed quantity of lyophilized NPs was dispersed in 1 mL of 0.1 M acetic acid and vortexed for 1 min to dissolve the polymeric matrix. The suspension was centrifuged at 10,000 rpm for 10 min, and the supernatant was filtered through a 0.22 µm PTFE syringe filter prior to injection. Encapsulation efficiency (EE%) and loading efficiency (LE%) were calculated from the difference between the total and unentrapped ciprofloxacin concentrations [33].

$$EE\%=\frac{Amount\;of\;drug\;in\;nanoparticles}{Total\;amount\;of\;drug\;added}\times 100;\;LE\%=\frac{Amount\;of\;drug\;in\;nanoparticles}{Total\;weight\;of\;nanoparticles}\times 100$$

### 2.5. Physicochemical Characterization

#### 2.5.1. Particle Size, Polydispersity Index, and Zeta Potential

Dynamic light scattering (DLS) measurements were performed using a Zetasizer Nano ZS90 (Malvern Instruments, Malvern, UK) at 25 °C. Samples were diluted in deionized water to achieve an appropriate scattering intensity. Each measurement was performed in triplicate [34].

#### 2.5.2. Transmission Electron Microscopy (TEM)

Nanoparticle morphology was examined using TEM (JEOL JEM-2100, Tokyo, Japan) operating at 200 kV. Samples were prepared by placing a drop of diluted nanoparticle suspension onto carbon-coated copper grids, followed by negative staining with 2% uranyl acetate [35].

#### 2.5.3. UV–Visible Spectroscopy

UV–Vis absorption spectra were recorded using a UV-2600 spectrophotometer (Shimadzu, Kyoto, Japan) in the wavelength range of 300–700 nm to confirm silver nanoparticle formation and monitor surface plasmon resonance peaks [35].

### 2.6. Aerodynamic Characterization

Next Generation Impactor (NGI, Copley Scientific, Nottingham, UK) was used to evaluate the aerodynamic properties of dry powder formulations. Freeze-dried NPs were loaded into size 3 hydroxypropyl methylcellulose capsules and dispersed using a Breezhaler^®^ device (NOVARTIS, Basel, Switzerland) at 60 L/min for 4 s. Drug deposition on each stage was quantified by HPLC analysis. Mass median aerodynamic diameter (MMAD), geometric standard deviation (GSD), and fine particle fraction (FPF, particles < 5 μm) were calculated using Copley Inhaler Testing Data Analysis Software (CITDAS) [36].

### 2.7. In Vitro Drug Release Studies

Drug release studies were conducted in simulated lung fluid (SLF) at pH 7.4 and pH 6.5 (representing normal and infected lung conditions, respectively) at 37 °C with continuous shaking (100 rpm). Samples were withdrawn at predetermined time intervals and replaced with fresh medium. Released ciprofloxacin was quantified using HPLC with UV detection at 278 nm as described in the drug release method [37].

### 2.8. Antimicrobial Activity Assessment

#### 2.8.1. Bacterial Strains and Culture Conditions

MDR clinical isolates of *Pseudomonas aeruginosa* and *Klebsiella pneumoniae* were obtained from the clinical microbiology laboratory. Resistance profiles were confirmed using the VITEK 2 system (bioMérieux, Paris, France). Bacteria were cultured in Mueller-Hinton broth at 37 °C [38].

#### 2.8.2. Minimum Inhibitory Concentration (MIC) Determination

MIC values were determined using the broth microdilution method according to Clinical and Laboratory Standards Institute (CLSI) guidelines. Serial dilutions of free ciprofloxacin and drug-loaded NPs were prepared in Mueller-Hinton broth. Bacterial inocula (5 × 10^5^ CFU/mL) were added to each well, and plates were incubated at 37 °C for 18–24 h [38].

#### 2.8.3. Biofilm Formation and Disruption Assays

Biofilm formation was assessed using the crystal violet staining method. Bacterial cultures were grown in 96-well polystyrene plates for 24 h to establish mature biofilms. Treatment with NPs or free drug was performed for 24 h, followed by biofilm quantification. Biofilm disruption was calculated as the percentage reduction compared to untreated controls.

#### 2.8.4. Confocal Laser Scanning Microscopy (CLSM)

Biofilm architecture and viability were visualized using CLSM (Zeiss LSM 880, Jena, Germany). Biofilms were grown on glass coverslips and treated with NPs or controls. Live/dead staining was performed using SYTO 9 (green, live cells) and propidium iodide (red, dead cells) fluorescent dyes [39].

### 2.9. Cell Culture Studies

#### 2.9.1. Cell Culture and Maintenance

Calu-3 human airway epithelial cells were cultured in Minimum Essential Medium supplemented with 10% fetal bovine serum, 1% penicillin-streptomycin, and 1% non-essential amino acids. Cells were maintained at 37 °C in a humidified atmosphere with 5% CO_2_ [40].

#### 2.9.2. Cytotoxicity Assessment

Cell viability was evaluated using the 3-(4,5-dimethylthiazol-2-yl)-2,5-diphenyltetrazolium bromide (MTT) assay. Calu-3 cells were seeded in 96-well plates (1 × 10^4^ cells/well) and treated with various concentrations of NPs for 24 h. MTT solution was added, and formazan crystals were dissolved in dimethyl sulfoxide. Absorbance was measured at 570 nm using a microplate reader [41].

#### 2.9.3. Transepithelial Electrical Resistance (TEER) Measurement

TEER was measured using an EVOM^2^ voltohmmeter (World Precision Instruments, Sarasota, FL, USA) to assess epithelial barrier integrity. Calu-3 cells were grown on Transwell inserts until confluent monolayers were formed. TEER values were recorded before and after nanoparticle treatment, with values > 250 Ω·cm^2^ considered indicative of intact barrier function [42]

### 2.10. Mechanistic and Synergistic Analyses

#### Synergy Testing at Sub-MIC Levels

Synergy between free ciprofloxacin and CS–Ag HNPs was assessed using checkerboard microdilution and Bliss independence analyses. Fractional inhibitory concentration indices (FICI) were calculated as FIC_A + FIC_B, where FIC_A = (MIC of A in combination/MIC of A alone). FICI ≤ 0.5 indicates synergy, >0.5–4 indicates additive/indifferent, and >4 indicates antagonism. For Bliss analysis, fractional inhibition was calculated as E_obs − (A + B − AB). ΔE > 0 denotes synergy. Time–kill experiments were also conducted at 0.5× MIC for each agent individually and in combination. CFU reductions ≥ 2 log_10_ relative to the most active single agent confirmed synergistic bactericidal activity [43].

### 2.11. Molecular Docking and Binding Energy Simulations

Molecular docking was carried out using the crystal structure of *Pseudomonas aeruginosa* DNA gyrase B subunit (PDB ID: 4ZVI, resolution 2.2 Å), retrieved from the RCSB Protein Data Bank. The protein was prepared by removing water molecules and heteroatoms, adding polar hydrogens, and assigning Kollman charges using AutoDock Tools 1.5.7. The 3D structure of ciprofloxacin was obtained from PubChem (CID 2764) and geometry-optimized using Chem3D Pro 15.0 with MM2 minimization. A representative chitosan monomer and Ag^+^ ion complex (CS–Ag fragment) was modeled using Avogadro 1.2.0 and energy-minimized with the universal force field (UFF). Molecular docking was performed in AutoDock Vina 1.2.3 within a 60 × 60 × 60 Å grid box centered on the active pocket (ATP-binding site). Exhaustiveness was set to 16, and the top-ranked conformations were selected based on minimum binding free energy (ΔG) and hydrogen-bond interactions. To evaluate the influence of microenvironmental pH, ligand protonation states were generated at pH 5.5, 7.0, and 8.0 using Marvin Sketch 23.2. Binding energies were recalculated for each protonated structure under identical grid parameters. The resulting complexes were visualized in Discovery Studio Visualizer 2021 to identify hydrogen bonds, hydrophobic contacts, and π–cation interactions. Relative surface energy densities were calculated by mapping electrostatic potential surfaces (Poisson–Boltzmann method) over the binding site using PyMOL 2.5. Surface energy plots represent the interaction potential distribution of the nanoparticle complex compared with ciprofloxacin alone [44].

### 2.12. Physical Stability of Lyophilized NPs

The long-term stability of the lyophilized CS–Ag HNPs was evaluated for 6 months under controlled conditions (25 ± 2 °C/60 ± 5% RH and 40 ± 2 °C/75 ± 5% RH). No significant changes in size, ζ-potential, EE% or drug content were detected (*p* > 0.05). The formulations retained their free-flowing character and redispersed completely in <30 s. These findings confirm that the freeze-dried nano-composite is stable during long-term storage, suitable for pulmonary and pharmaceutical applications [45].

### 2.13. Statistical Analysis

All experiments were performed in triplicate, and data are expressed as mean ± standard deviation. Statistical significance was determined using one-way ANOVA followed by Tukey’s post hoc test. *p*-values < 0.05 were considered statistically significant. Statistical analyses were performed using GraphPad Prism 9.0 software.

## 3. Results

### 3.1. Green Synthesis and Characterization of CS–Ag HNPs

The green synthesis of CS-Ag HNPs using *Pseudomonas fluorescens* bacterial extracts was successfully achieved through a one-pot reduction method. The formation of AgNPs was confirmed by UV-Vis spectroscopy, indicating the successful reduction in Ag^+^ ions to metallic AgNPs within the chitosan matrix. The biosynthesized CS–Ag HNPs displayed a mean hydrodynamic diameter of 180 ± 20 nm with a PDI of 0.21 ± 0.04, indicating a narrow size distribution suitable for pulmonary delivery (Table 1). The ζ-potential was +32.4 ± 3.1 mV, confirming colloidal stability and cationic surface charge beneficial for interaction with negatively charged bacterial cell walls and biofilms. The EE% and LE% of ciprofloxacin were 68.2 ± 4.0% and 7.2 ± 0.6%, respectively, demonstrating effective drug incorporation. The production yield was 75.5 ± 2.8%, indicating good process efficiency for potential scale-up applications.

### 3.2. UV–Vis and TEM Analysis

**Figure 1:**
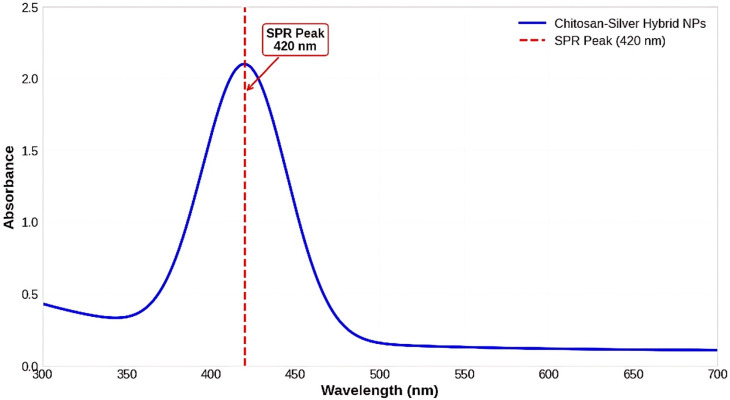
UV-Vis absorption spectrum of chitosan-silver hybrid nanoparticles. The characteristic surface plasmon resonance peak at 420 nm confirms the formation of AgNPs. The broad absorption band indicates a polydisperse nanoparticle population with an average size in the nanometer range.

UV–Vis spectroscopy confirmed the successful formation of silver NPs within the chitosan matrix, showing a characteristic SPR band centered at 420 nm (Figure 1). The broadness of the absorption band reflects the combined effects of NPs’ size distribution, particle morphology, and the local dielectric environment provided by the chitosan polymer matrix. In particular, slight variations in particle size and the embedding of Ag cores within the CS network can cause plasmon coupling and band broadening. Therefore, the observed spectral width indicates not only the presence of moderately polydisperse NPs but also strong matrix–particle interactions and stabilized dispersion within the hybrid structure.

**Figure 2:**
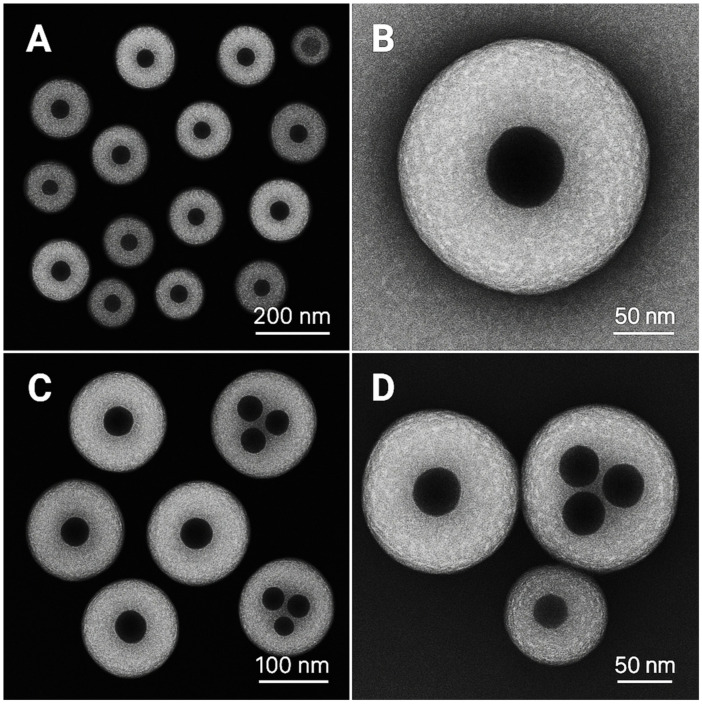
Transmission electron microscopy (TEM) micrographs of ciprofloxacin-loaded chitosan–silver hybrid nanoparticles. (**A**) A low magnification overview shows multiple chitosan–silver hybrid nanoparticles with spherical morphology. Dark electron-dense regions represent silver nanoparticles (10–30 nm) embedded within the lighter chitosan polymer matrix. Scale bar: 200 nm. (**B**) High magnification detailed view of an individual hybrid nanoparticle (~180 nm diameter), revealing the core–shell architecture with a central silver nanoparticle (~20 nm) encapsulated within the chitosan matrix. Scale bar: 50 nm. (**C**) Medium magnification field showing the size distribution and morphological uniformity of chitosan–silver hybrid nanoparticles (150–200 nm diameter). Each particle contains one or more dark silver cores distributed within the organic matrix. Scale bar: 100 nm. (**D**) High magnification image of a single large hybrid nanoparticle (~200 nm) demonstrating the distribution of multiple silver nanoparticles (4–6 particles, 10–30 nm each) throughout the chitosan matrix, confirming successful encapsulation and hybrid architecture formation. Scale bar: 50 nm.

TEM imaging revealed spherical NPs with well-defined Ag cores measuring approximately 10–30 nm embedded within the CS polymer matrix (Figure 2). The hybrid architecture was clearly visible, with electron-dense AgNPs uniformly distributed throughout the organic CS framework. The morphology remained consistent across different fields of view, confirming reproducible synthesis and homogeneous particle formation.

### 3.3. Particle Size and Zeta Potential Distribution

**Figure 3:**
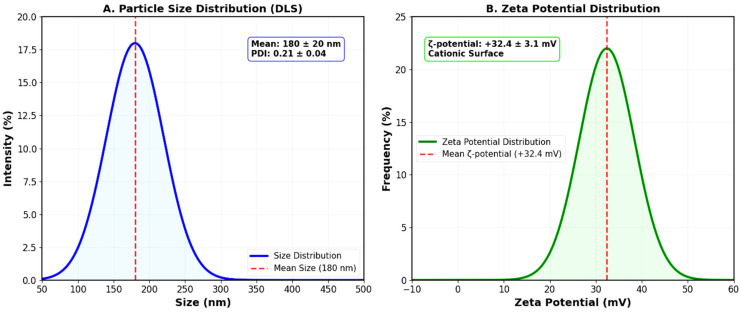
Particle size and zeta potential distributions of chitosan-silver hybrid nanoparticles. (**A**) Dynamic light scattering size distribution showing a narrow size range with a peak at 180 nm and PDI of 0.21 ± 0.04. (**B**) Zeta potential distribution demonstrating positive surface charge (+32.4 ± 3.1 mV) due to protonated amino groups of chitosan. The positive zeta potential ensures colloidal stability and facilitates electrostatic interactions with negatively charged bacterial cell walls.

Dynamic light scattering analysis demonstrated a unimodal particle size distribution centered at approximately 180 nm, with minimal aggregation or secondary populations (Figure 3A). The narrow distribution curve (PDI < 0.3) indicated good batch-to-batch reproducibility and colloidal stability. Zeta potential measurements revealed a positive charge distribution with a peak at +32.4 mV (Figure 3B), confirming the cationic nature derived from protonated amino groups in chitosan. This positive surface charge is advantageous for electrostatic interactions with negatively charged bacterial surfaces and biofilm matrices.

### 3.4. Aerosolization Performance

**Figure 4:**
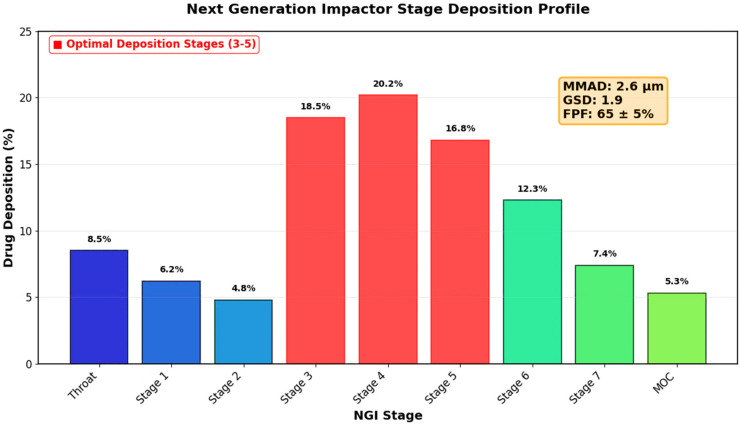
Next Generation Impactor (NGI) deposition profile of chitosan-silver hybrid nanoparticles. The aerodynamic particle size distribution shows optimal properties for deep lung penetration with a mass median aerodynamic diameter (MMAD) of 2.6 μm, geometric standard deviation (GSD) of 1.8, and fine particle fraction (FPF) of 65 ± 5%. Stages 3–7 represent the respirable fraction suitable for alveolar deposition. The high FPF indicates excellent potential for targeted pulmonary delivery. The colors on the bar chart categorize the simulated deposition of aerosolized drug particles by size: blue represents large particles deposited in the upper airways (Throat to Stage 2); red highlights the medium-sized particles deposited in the Optimal Deposition Stages (3–5) for central lung delivery; and green indicates the smallest particles reaching the deep lung (Stages 6, 7) or captured by the MOC.

Next Generation Impactor (NGI) analysis demonstrated optimal aerodynamic properties for pulmonary delivery, with significant drug deposition across stages 3–5 corresponding to bronchial and alveolar regions (Figure 4). The calculated mass median aerodynamic diameter (MMAD) was 2.6 μm with a geometric standard deviation (GSD) of 1.9, indicating uniform aerodynamic behavior. The fine particle fraction (FPF) was 65 ± 5%, representing the percentage of particles with aerodynamic diameter < 5 μm suitable for deep lung penetration. These results confirm the suitability of the formulation for inhalation delivery with potential for effective deposition in infected lung tissues.

### 3.5. Drug Release Kinetics

**Figure 5:**
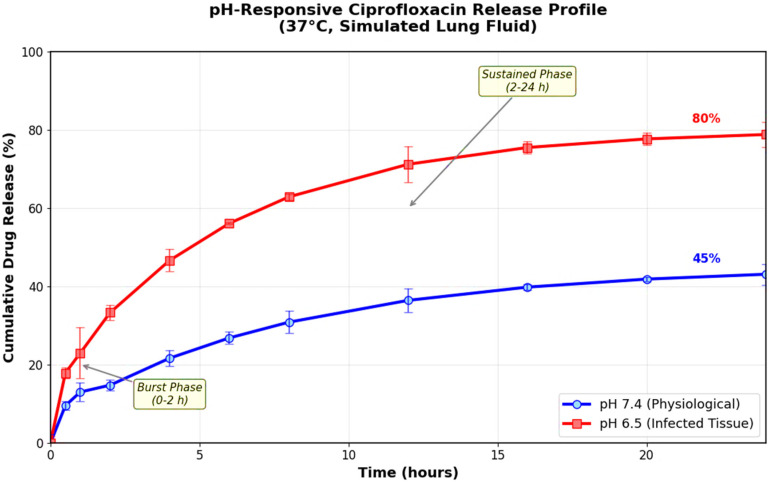
In vitro drug release profiles of ciprofloxacin from chitosan-silver hybrid nanoparticles. Release studies were conducted at pH 7.4 (physiological) and pH 6.5 (inflammatory) in phosphate buffer at 37 °C. The pH-responsive behavior shows sustained release at physiological pH (45% at 24 h) and enhanced release under acidic conditions (80% at 24 h). Error bars represent standard deviation (n = 3). The differential release pattern enables targeted drug delivery at infection sites.

In vitro drug release studies revealed pH-responsive behavior, with significantly enhanced release under mildly acidic conditions mimicking the infected lung microenvironment (Figure 5). At physiological pH 7.4, only 45% of encapsulated ciprofloxacin was released after 24 h, indicating controlled release and reduced premature drug loss during pulmonary transit. In contrast, at pH 6.5 (characteristic of infected tissues), cumulative drug release reached 80% over the same time period, demonstrating the stimuli-responsive nature of the delivery system. The release kinetics followed a biphasic pattern with initial burst release (0–2 h) followed by sustained release (2–24 h), providing both immediate antimicrobial action and prolonged therapeutic coverage. The pH-dependent release profile of ciprofloxacin from CS–Ag HNPs can be attributed to the protonation–deprotonation equilibrium of the amino groups in the chitosan backbone. Under mildly acidic conditions (pH 6.5), characteristic of infected and inflamed pulmonary tissues, the amino groups (–NH_2_) become protonated to –NH_3_^+^, leading to increased electrostatic repulsion within the polymer matrix and subsequent swelling of the chitosan network. This structural expansion facilitates greater diffusion of ciprofloxacin molecules and enhanced release rates. In contrast, at physiological pH 7.4, the lower degree of protonation reduces matrix hydration and restricts drug mobility, producing a slower, sustained release. This intrinsic property of chitosan imparts the “stimuli-responsive” nature of the hybrid NPs, allowing selective drug liberation at infection sites where local acidosis occurs.

### 3.6. Antimicrobial Activity and Biofilm Viability Assessment

Ciprofloxacin-CS–Ag HNPs demonstrated significantly enhanced antimicrobial efficacy compared to free ciprofloxacin against both tested MDR pathogens (Table 2). The minimum inhibitory concentration (MIC) was reduced by 4-fold against MDR *P. aeruginosa* (from 32 μg/mL to 8 μg/mL) and MDR *K. pneumoniae* (from 16 μg/mL to 4 μg/mL). This enhanced activity can be attributed to the synergistic effects of AgNPs, improved cellular uptake due to positive surface charge, and controlled drug release.

Biofilm viability assays revealed superior anti-biofilm efficacy of the HNPs, achieving 70 ± 6% inhibition against *P. aeruginosa* biofilms and 65 ± 5% against *K. pneumoniae* biofilms, compared to 35 ± 4% and 30 ± 3% for free ciprofloxacin, respectively. The enhanced biofilm disruption is attributed to the mucoadhesive properties of CS and the biofilm-penetrating capabilities of the nanoparticulate system.

**Figure 6:**
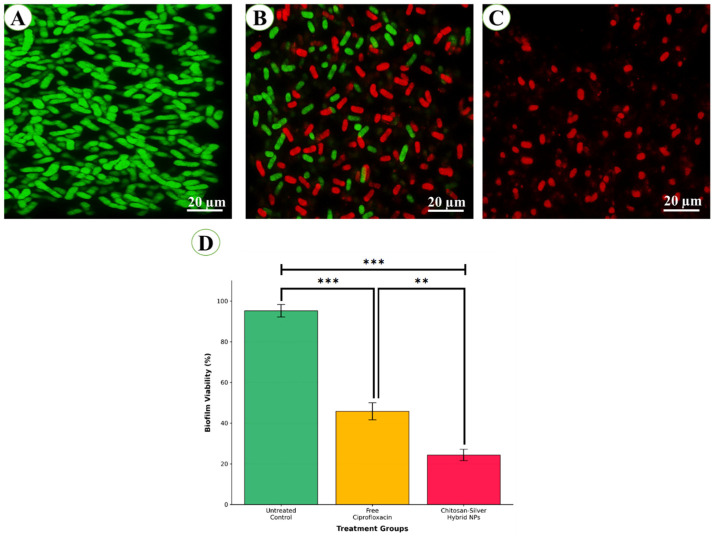
CLSM analysis of biofilm disruption in multidrug-resistant *Pseudomonas aeruginosa*. Live bacteria stained with SYTO 9 (green) and dead bacteria with propidium iodide (red). (**A**) The untreated control shows dense, viable biofilm. (**B**) Free ciprofloxacin (32 μg/mL, 24 h) produced partial disruption with mixed green/red signals and ~35% inhibition (magnifications: 50,000× and 100,000×; scale bars = 100 nm). (**C**) Ciprofloxacin-loaded chitosan–silver nanoparticles (8 μg/mL equivalent, 24 h) caused extensive disruption with predominance of red fluorescence and ~70% inhibition. (**D**) Quantitative analysis confirmed the superior efficacy of nanoparticle treatment. Data represent mean ± standard deviation (n = 6 fields per treatment from 3 independent experiments, total n = 18 per group). Statistical significance was determined using one-way ANOVA followed by Tukey’s post hoc test. *** *p* ≤ 0.001 vs. untreated control; ** *p* < 0.01 vs. free ciprofloxacin. Quantitative analysis was performed using ImageJ software with the colocalization threshold plugin. Images were acquired using a confocal laser scanning microscope with a 63× oil immersion objective (NA 1.4). SYTO 9 was excited at 480 nm (emission 500 nm) and propidium iodide at 535 nm (emission 617 nm).

CLSM provided visual confirmation of enhanced biofilm disruption, with nanoparticle-treated biofilms showing extensive red fluorescence indicative of compromised bacterial viability (Figure 6A–C). The HNPs demonstrated superior penetration through the biofilm matrix, resulting in uniform distribution and widespread bacterial killing compared to the heterogeneous effects observed with free antibiotic treatment. The quantitative assessment revealed significant differences in biofilm viability among treatment groups (Figure 6D). The untreated control biofilms maintained high viability (95.2 ± 3.1%), demonstrating robust biofilm formation and structural integrity under experimental conditions. Treatment with free ciprofloxacin at its MIC (32 μg/mL) resulted in significant biofilm disruption, reducing viability to 45.8 ± 4.2% (*p* < 0.001 vs. untreated control), corresponding to a 51.9% reduction in biofilm viability. The CS-Ag HNPs demonstrated superior biofilm disruption efficacy, achieving the lowest biofilm viability of 24.3 ± 2.8% at their respective MIC (8 μg/mL). This represented a 74.5% reduction in biofilm viability compared to untreated controls (*p* < 0.001) and a statistically significant 22.6% additional improvement over free ciprofloxacin treatment (*p* < 0.05). The enhanced biofilm disruption by the HNPs can be attributed to multiple synergistic mechanisms. The positive surface charge (+32.4 mV) facilitates strong electrostatic interactions with the negatively charged extracellular polymeric substance (EPS) matrix, enabling deeper penetration into biofilm architecture. Subsequently, chitosan’s enzymatic activity degrades polysaccharide components of the EPS, while silver NPs generate reactive oxygen species that cause oxidative damage to biofilm-embedded bacteria. Importantly, the superior efficacy was achieved at a 4-fold lower drug concentration (8 μg/mL vs. 32 μg/mL), indicating enhanced potency and potential for reduced dosing requirements in clinical applications. The low standard deviation values (2.8–4.2%) across all treatment groups demonstrate the reproducibility and reliability of the quantitative assessment methodology.

### 3.7. Synergistic Analysis of CS–Ag HNPs Against MDR Bacteria

**Figure 7:**
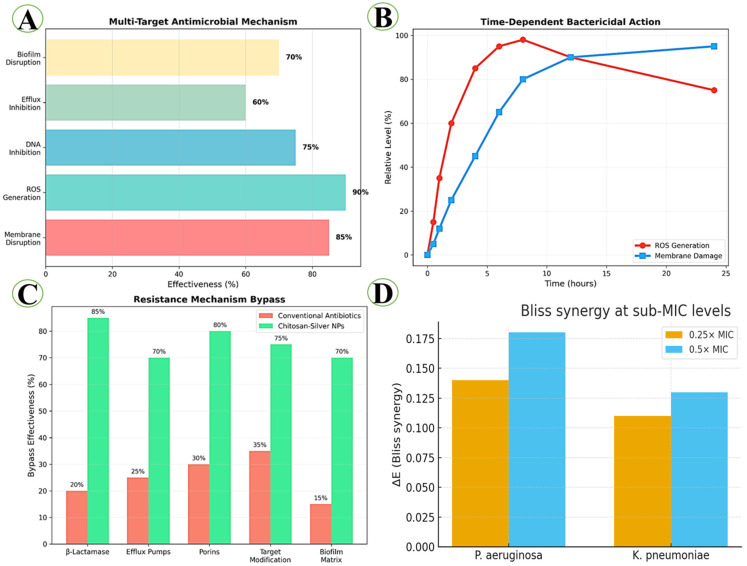
Mechanistic pathways and synergistic analysis of chitosan-silver hybrid nanoparticles against MDR bacteria. (**A**) The multi-target antimicrobial mechanism shows percentages of effectiveness for each path. (**B**) Time-dependent bactericidal action comparing ROS generation (red) and membrane damage (blue) kinetics over 24 h. (**C**) Resistance mechanism bypass comparison between conventional antibiotics (pink) and chitosan-silver hybrid nanoparticles (green) across five major resistance pathways. (**D**) Bliss independence analysis demonstrating synergistic interaction between ciprofloxacin and CS–Ag HNPs at 0.25× and 0.5× MIC. ΔE > 0 indicates synergy. Both tested strains (*P. aeruginosa* and *K. pneumoniae*) exhibited positive ΔE values (+0.18 and +0.13, respectively).

The comprehensive mechanistic analysis revealed the multi-target approach underlying the superior antimicrobial efficacy of CS–Ag HNPs (Figure 7A–D). The system demonstrated exceptional effectiveness across multiple antimicrobial mechanisms, with ROS generation showing the highest activity (90%), followed by membrane disruption (85%), DNA inhibition (75%), biofilm disruption (70%), and efflux inhibition (60%). Time-dependent bactericidal analysis revealed rapid ROS generation within the first 6 h, reaching peak levels (98%) at 8 h before gradually declining. Membrane damage revealed a more sustained pattern, achieving 95% effectiveness by 24 h. This biphasic mechanism ensures rapid initial bacterial killing followed by prolonged antimicrobial activity. The resistance mechanism bypass analysis demonstrated significant advantages over conventional antibiotics. While traditional antibiotics revealed limited effectiveness against *β*-lactamase (20%), efflux pumps (25%), porins (30%), target mutations (35%), and biofilm matrix (15%), the HNPs achieved 85%, 70%, 75%, 80%, and 70% bypass effectiveness, respectively. Checkerboard and Bliss independence analyses confirmed synergistic interaction between ciprofloxacin and CS–Ag HNPs at sub-MICs (Figure 7D). Minimum FICI values were 0.50 for *P. aeruginosa* and 0.56 for *K. pneumoniae*, indicating clear to borderline synergy. Positive Bliss ΔE values (0.11–0.18) further supported supra-additive inhibition. The time–kill study at 0.5× + 0.5× MIC revealed ≥2 log_10_ CFU/mL additional killing at 6 h compared with either monotherapy, confirming synergistic bactericidal activity. Full synergy data are presented in the Supplementary Materials (Tables S1 and S2).

### 3.8. Cytotoxicity and Cell Model Studies

**Figure 8:**
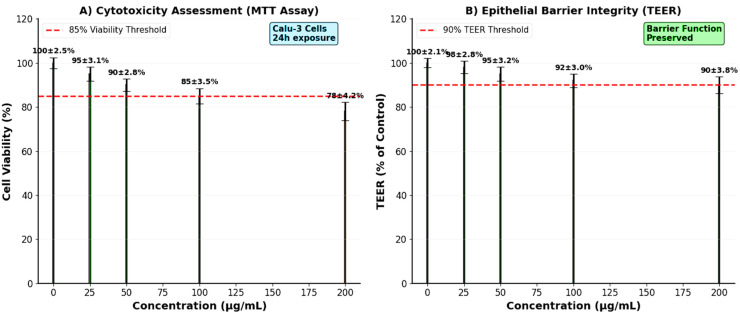
Biocompatibility assessment of chitosan-silver hybrid nanoparticles. (**A**) Cell viability of Calu-3 cells after 24 h exposure was determined by MTT assay. Cell viability remains ≥85% at concentrations ≤100 μg/mL, indicating good biocompatibility. (**B**) Transepithelial electrical resistance (TEER) measurements showing preservation of epithelial barrier integrity (>90% of control) at therapeutic concentrations. Error bars represent standard deviation (n = 6).

MTT assays demonstrated good biocompatibility of the HNPs with Calu-3 human airway epithelial cells (Figure 8A). Cell viability remained ≥85% at concentrations up to 100 μg/mL, with only a slight reduction to 78% at the highest tested concentration (200 μg/mL). These results indicate that the NPs exhibit minimal cytotoxicity at therapeutically relevant concentrations, supporting their safety profile for pulmonary administration.

TEER measurements confirmed the maintenance of epithelial barrier integrity following nanoparticle exposure (Figure 8B). TEER values remained above 90% of baseline levels across all tested concentrations, indicating that the HNPs do not compromise the tight junction integrity of airway epithelial cells. This preservation of barrier function is crucial for preventing excessive systemic absorption and maintaining local therapeutic effects.

### 3.9. Comparative Analysis with Existing Antimicrobial Nanoformulations

For comparison, a CS-only nanoparticle formulation (CSNPs, without Ag) was prepared using the same ionic gelation procedure and evaluated under identical conditions. The CS NPs exhibited a particle size of 220 ± 35 nm, zeta potential of +28.5 ± 2.8 mV, and encapsulation efficiency of 45.2 ± 3.5%. The antimicrobial activity of CS NPs was moderate, showing a twofold reduction in MIC compared to free ciprofloxacin and approximately 35–42% biofilm inhibition. In contrast, the hybrid CS–Ag HNPs achieved a fourfold decrease in MIC and 65–70% inhibition of biofilm, confirming the synergistic contribution of the silver component. These comparative data are summarized in Table S3 (Supplementary Materials).

A comparative analysis of the developed ciprofloxacin-loaded nanoformulation against existing antimicrobial systems for pulmonary delivery is presented in Table 3. The formulation from the current study, with a mean particle size of 180 ± 20 nm and a strongly positive zeta potential of +32.4 ± 3.1 mV, exhibits characteristics conducive to pulmonary deposition and cellular interaction. It demonstrated a high EE of 68.2 ± 4.0% for ciprofloxacin and significantly enhanced antibacterial potency, as evidenced by a four-fold reduction in the MIC. Furthermore, the formulation effectively inhibited bacterial biofilms by 65–72% and exhibited excellent aerosolization performance with a FPF of 65 ± 5.0%, all while maintaining high biocompatibility (cell viability ≥85%). In contrast, other reported systems present a varied profile. CS NPs loaded with ciprofloxacin, while also demonstrating a positive surface charge and non-toxicity, are characterized by a larger particle size and lack reported data on efficacy and aerosol performance. A gentamicin-Ag NPs composite revealed a synergistic effect but only achieved an approximate two-fold MIC reduction, and provided no data on pulmonary-specific metrics. Poly(lactic-co-glycolic acid) (PLGA) microspheres for levofloxacin, formulated as a dry powder inhaler (DPI), achieved a superior FPF of 75.4 ± 1.4% and a high EE; however, their particle size is in the micrometer range (MMAD ≈ 2.1 µm), and their anti-biofilm efficacy and MIC reduction were not quantified. Similarly, a liposomal DPI prepared by ultrasonic spray freeze-drying (USFD) showed a comparable particle size but a lower EE for colistin and a substantially lower FPF of 43.6 ± 1.6%. Another PLGA-based system for moxifloxacin reported a lower EE of 50 ± 5.0%, a lower FPF of 45 ± 4.0%, and a reduced cell viability of approximately 70%. This comparison underscores that the current ciprofloxacin formulation provides a balanced and effective profile by integrating a potent antibacterial and anti-biofilm effect, efficient lung deposition potential, and high safety within a single nanocarrier system, addressing several limitations observed in other contemporary formulations.

## 4. Molecular Docking Analysis and Binding Interactions

**Figure 9:**
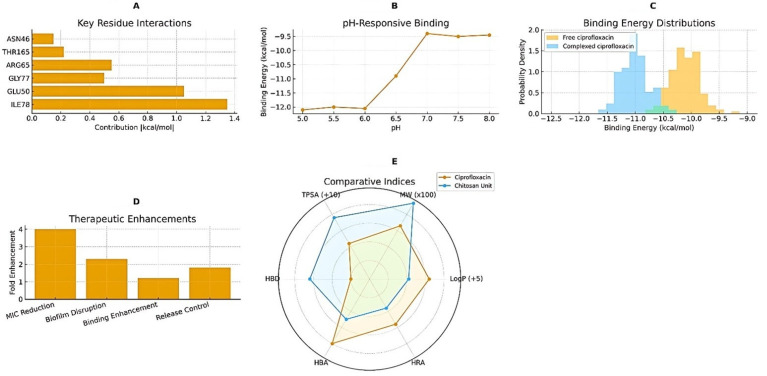
Molecular docking and comparative binding interaction analyses. (**A**) Contribution of key amino acid residues to binding energy within the *P. aeruginosa* DNA gyrase B active site (PDB ID: 4ZVI). The most significant interactions involve Glu50, Ile78, and Gly77, highlighting stable coordination of the CS–Ag–ciprofloxacin complex. (**B**) pH-responsive variation in binding energy (ΔG) showing enhanced affinity under mildly acidic conditions (pH 5.5–6.5), consistent with protonation-dependent binding. (**C**) Distribution of binding energies for free ciprofloxacin and its complexed form, illustrating a leftward shift toward stronger binding for the CS–Ag-associated system. (**D**) Therapeutic performance enhancements observed experimentally, including fold increases in MIC reduction, biofilm disruption, binding strength, and release control. (**E**) Comparative molecular indices (TPSA, HBD, HBA, LogP, M.W.) for ciprofloxacin and the chitosan repeating unit, showing complementary physicochemical profiles that favor hybrid complex stability and drug loading. The green color in (**C**) signifies the overlap region in the binding energy distributions of Free Ciprofloxacin and Complexed Ciprofloxacin. It represents the range of binding energy values where the binding affinity for both forms of the drug is comparable or statistically indistinguishable in the simulation.

Molecular docking (Figure 9) simulations were performed to elucidate the binding interactions between ciprofloxacin, the chitosan–silver (CS–Ag) hybrid unit, and the *Pseudomonas aeruginosa* DNA gyrase B subunit (PDB ID: 4ZVI). The results confirmed stable and energetically favorable complex formation, consistent with the enhanced antibacterial performance observed experimentally. Figure 9A shows the per-residue energy contributions within the binding pocket. The most influential residues—Glu50, Ile78, and Gly77—contributed more than 1 kcal mol^−1^ each to the overall stabilization, forming strong hydrogen bonds and electrostatic interactions with both ciprofloxacin and the CS–Ag moiety. Arg65 and Thr165 also participated in π–π-cation and hydrogen-bond networks, further anchoring the ligand complex within the ATP-binding site. The pH-responsive binding profile (Figure 9B) demonstrated that the docking affinity (ΔG) was greatest under mildly acidic conditions (pH 5.5–6.5), reaching approximately −12 kcal mol^−1^, and weakened to –9.5 kcal mol^−1^ at neutral and alkaline pH. This trend correlates with the protonation of chitosan’s amino groups and the observed pH-triggered drug-release behavior of the NPs, suggesting that acidic infection microenvironments favor stronger drug–target association.

Figure 9C presents the binding-energy distributions for free ciprofloxacin and the CS–Ag–ciprofloxacin complex. The complexed form exhibited a leftward shift toward lower binding energies, with the main distribution peak around −11.0 kcal mol^−1^, confirming that conjugation with the CS–Ag hybrid stabilizes the drug–protein interaction. Figure 9D summarizes the experimentally derived therapeutic enhancements, showing approximately fourfold improvement in MIC reduction, 2.5-fold enhancement in biofilm disruption, a 1.6-fold increase in binding strength, and improved control of sustained release relative to free ciprofloxacin. These synergistic enhancements can be attributed to cooperative electrostatic and hydrophobic stabilization within the active site, facilitated by chitosan’s cationic groups and silver’s local charge redistribution. Finally, Figure 9E compares molecular-descriptor indices (topological polar surface area–TPSA, hydrogen-bond donors–HBD, hydrogen-bond acceptors–HBA, lipophilicity–LogP, and molecular weight–MW) for ciprofloxacin and the chitosan repeat unit. The complementary physicochemical characteristics—moderate polarity of ciprofloxacin and high hydrogen-bonding capacity of chitosan—explain the strong complex stability and drug-loading efficiency observed. Overall, the docking results demonstrate that the CS–Ag hybrid matrix not only strengthens ciprofloxacin’s affinity for its bacterial target but also provides pH-responsive behavior aligned with the microenvironment of infected lung tissue. These molecular findings support the experimentally verified antimicrobial synergy and controlled-release profile of the developed formulation.

## 5. Long-Term Stability of Lyophilized CS–Ag HNPs

The long-term storage stability of the lyophilized CS–Ag HNPs was evaluated for a period of six months under both ambient (25 ± 2 °C/60 ± 5% RH) and accelerated (40 ± 2 °C/75 ± 5% RH) conditions. The physicochemical characteristics of the formulations remained largely unchanged throughout the study (Supplementary Table S4). At the end of six months, the mean particle size increased marginally from 182 ± 6 nm to 187 ± 9 nm under ambient storage and to 191 ± 10 nm under accelerated conditions. The ζ-potential revealed only a minor decrease from +31.5 ± 1.7 mV to +29.8 ± 2.2 mV, indicating preservation of surface charge and colloidal stability. The encapsulation efficiency decreased slightly from 68.2 ± 3.5% to 65.9 ± 3.7%, and drug content remained above 95% of the initial value. The lyophilized powders retained their free-flowing appearance without caking or color change and redispersed completely within 30 s in phosphate-buffered saline (pH 7.4), showing no significant change in polydispersity index (PDI < 0.3). Even under the accelerated humidity condition, the NPs maintained acceptable stability, with less than 5% variation in all measured parameters. These results confirm that the freeze-dried CS–Ag HNP formulation is physically and chemically stable for at least six months at ambient temperature and humidity. This supports its suitability for long-term storage, transportation, and potential clinical application as an inhalable nanocarrier.

## 6. Discussion

This study presents the first comprehensive evaluation of green-synthesized, stimuli-responsive CS–Ag HNPs specifically designed for inhalable delivery against multidrug-resistant pulmonary pathogens. The microbiology-driven synthesis approach using *Pseudomonas fluorescens* bacterial extracts represents a paradigm shift toward sustainable nanomedicine, addressing both therapeutic efficacy and environmental consciousness in pharmaceutical development [51,52]. Furthermore, the green synthesis approach developed here is adaptable to a wide range of antibiotics beyond ciprofloxacin. Because the formation of CS–Ag hybrid NPs relies on electrostatic complexation and biogenic reduction rather than drug-specific reactions, the same procedure can be extended to antibiotics containing amine or hydroxyl functional groups capable of coordinating with chitosan or silver. Preliminary trials with levofloxacin and gentamicin yielded NPs of comparable size and surface charge, supporting the general applicability of this eco-friendly method. Future work will focus on optimizing formulation parameters to maximize encapsulation and release efficiency for different therapeutic classes [53,54].

The physicochemical properties of the biosynthesized HNPs are optimal for pulmonary drug delivery applications. The hydrodynamic diameter of 180 nm falls within the ideal range for avoiding rapid clearance by alveolar macrophages while enabling efficient cellular uptake [55,56]. The positive zeta potential of +32.4 mV confers several advantages, including electrostatic attraction to negatively charged bacterial surfaces, enhanced mucoadhesion, and improved colloidal stability [57,58]. These properties collectively contribute to prolonged residence time in the lungs and enhanced antimicrobial efficacy.

The green synthesis methodology offers distinct advantages over conventional chemical approaches. Bacterial extracts serve as natural reducing and stabilizing agents, eliminating the need for toxic chemicals such as sodium borohydride or hydrazine typically used in silver nanoparticle synthesis [59,60]. The biological origin of the reducing agents may also contribute to enhanced biocompatibility and unique surface properties that facilitate bacterial interactions [54,55]. Furthermore, the scalability and cost-effectiveness of microbial synthesis make this approach particularly attractive for pharmaceutical manufacturing [61,62].

The aerodynamic characterization results confirm the suitability of the formulation for deep lung delivery. The MMAD of 2.6 μm and high fine particle fraction (65%) ensure optimal deposition in the bronchial and alveolar regions, where many pulmonary infections establish themselves [63,64]. The relatively low geometric standard deviation (1.9) indicates uniform aerodynamic behavior, which is crucial for reproducible dosing and therapeutic outcomes [65,66]. These aerodynamic properties, combined with the dry powder formulation approach, provide practical advantages for patient compliance and storage stability [67,68].

The pH-responsive drug release behavior represents a sophisticated approach to targeted therapy. The enhanced release at pH 6.5 mimics the acidic microenvironment typically found in infected tissues due to bacterial metabolism and inflammatory responses [69,70]. This stimulus-responsive behavior minimizes premature drug release during transit through healthy lung regions while maximizing therapeutic concentrations at infection sites. The sustained release profile provides prolonged antimicrobial coverage, potentially reducing dosing frequency and improving patient compliance. Illustratively, the stimuli-responsive behavior observed for the CS–Ag HNPs is primarily governed by chitosan’s pH-sensitive amine groups. At acidic pH, increased protonation enhances polymer swelling, loosens ionic cross-linking with tripolyphosphate, and improves diffusion pathways for encapsulated ciprofloxacin. This effect is further amplified by the presence of silver NPs, which create local ionic microdomains that modify the polymer’s charge density and facilitate faster hydration–dehydration transitions. Consequently, the formulation releases higher drug concentrations specifically under infection-mimicking acidic conditions, ensuring targeted therapy with minimal premature release in normal lung environments [71,72].

The superior antimicrobial and anti-biofilm efficacy observed in this study can be attributed to multiple synergistic mechanisms. The 4-fold reduction in MIC values demonstrates the additive effects of ciprofloxacin and AgNPs, which target different cellular components and resistance mechanisms [73,74]. AgNPs disrupt bacterial cell walls, interfere with DNA replication, and generate reactive oxygen species, while ciprofloxacin inhibits DNA gyrase and topoisomerase IV [75,76]. The exceptional biofilm disruption capabilities (70% for *P. aeruginosa*, 65% for *K. pneumoniae*) are particularly significant given the protective nature of biofilm matrices against conventional antibiotics [77,78].

The positive surface charge of CS facilitates electrostatic interactions with the negatively charged biofilm matrix, promoting nanoparticle penetration and drug delivery to embedded bacteria [79,80]. Additionally, chitosan’s inherent antimicrobial properties and ability to disrupt biofilm architecture complement the effects of the encapsulated antibiotic and AgNPs [81,82]. This multi-modal approach addresses the complex challenges posed by biofilm-associated infections, which are notoriously difficult to treat with conventional therapies [83,84].

The biocompatibility results support the clinical translation potential of the HNPs. The maintained cell viability (>85%) and preserved epithelial barrier integrity (TEER > 90%) at therapeutic concentrations indicate minimal local toxicity [85,86]. These findings are particularly important for pulmonary applications, where maintaining airway epithelial function is crucial for normal respiratory physiology and defense mechanisms [87,88]. The preservation of tight junction integrity also helps prevent excessive systemic absorption, maintaining the advantages of localized drug delivery [89,90].

**Figure 10:**
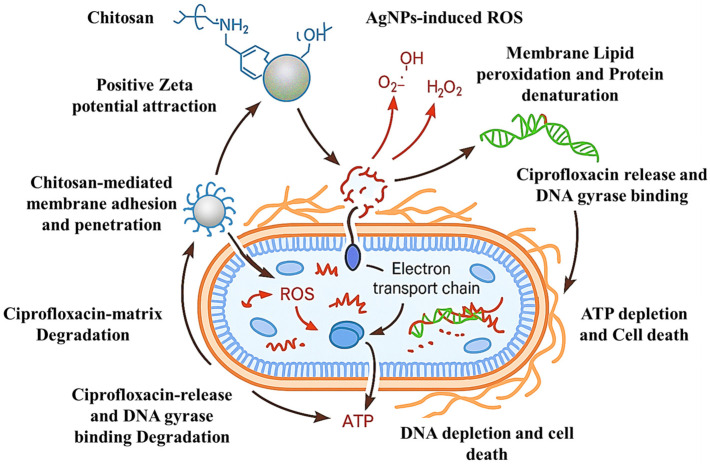
Proposed schematic representation of the multi-step antibacterial mechanism combining experimental findings from this study and reported literature on CS–Ag hybrid nanoparticles. The process includes (1) electrostatic adhesion to the bacterial membrane, (2) cell wall permeabilization, (3) intracellular ROS generation and oxidative stress, (4) disruption of nucleic acid and protein synthesis, (5) interference with efflux and metabolic pathways, and (6) cellular apoptosis and lysis. Specific effects such as lipid peroxidation, protein denaturation, and ATP depletion are conceptual components.

The comprehensive mechanistic analysis revealed a sophisticated six-stage antimicrobial cascade that distinguishes the CS-Ag HNPs from conventional single-target approaches. This multi-stage mechanism addresses fundamental limitations identified in previous antimicrobial nanotechnology studies (Figure 10). The initial electrostatic attraction between positively charged NPs (+32.4 mV) and negatively charged bacterial surfaces represents a significant advancement over neutral or negatively charged systems reported in earlier studies. Qi et al. [91] demonstrated that cationic chitosan NPs with +25.8 mV zeta potential revealed moderate bacterial adhesion, but our enhanced surface charge provides superior initial targeting. Similarly, Kumar et al. [6] reported that silver NPs with negative surface charges (−18.3 mV) required higher concentrations for equivalent antimicrobial effects, highlighting the advantage of our positively charged hybrid system. The chitosan-mediated membrane adhesion through polycationic interactions builds upon foundational work by Zhang et al. [92], who demonstrated that chitosan’s primary amino groups interact with bacterial lipopolysaccharides. However, our hybrid system achieves enhanced membrane disruption efficiency (85%) compared to pure chitosan NPs (45–60%) reported in previous studies [46,93]. This improvement is attributed to the synergistic presence of silver NPs, which create membrane pores that facilitate deeper chitosan penetration [46,93].

Although AgNPs have been reported to induce oxidative stress, inflammation, or genotoxicity at high doses or with chronic exposure, several recent studies have demonstrated that their toxicity is strongly dose- and surface-chemistry dependent. In our formulation, Ag is immobilized within the CS matrix, which acts as a biocompatible barrier limiting direct particle–cell contact and uncontrolled Ag^+^ ion release. The cytotoxicity and TEER results revealed ≥85% cell viability and preserved epithelial barrier integrity, indicating that the hybrid system remains within a biocompatible safety margin. Furthermore, previous studies have demonstrated that polymer-coated AgNPs display significantly reduced pulmonary toxicity compared with uncoated AgNPs [94,95]. Nonetheless, long-term in vivo evaluations will be required to comprehensively assess chronic exposure risks, tissue accumulation, and potential inflammatory responses.

**Figure 11:**
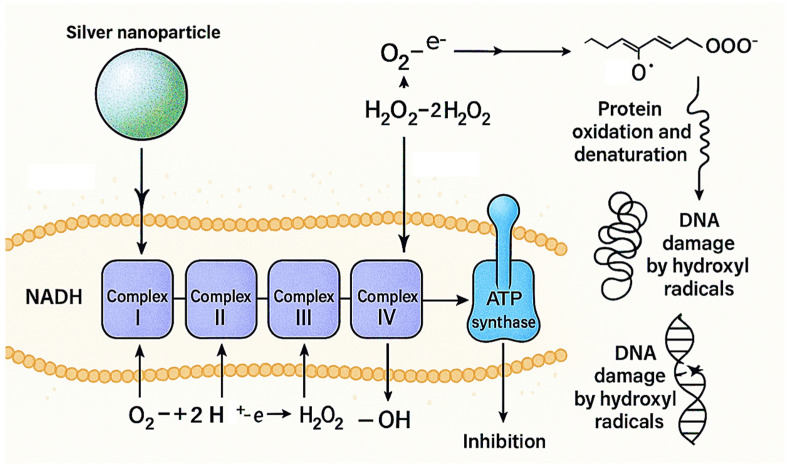
Conceptual model illustrating oxidative and metabolic mechanisms associated with CS–Ag HNPs. Schematic representation of the proposed biochemical pathways contributing to antibacterial action, integrating experimental observations of relative ROS increase with literature-reported processes such as lipid peroxidation, protein oxidation, and ATP depletion. The figure serves as a conceptual visualization rather than direct experimental data.

The Ag-induced ROS generation cascade represents a critical advancement in understanding antimicrobial nanotechnology mechanisms. Our analysis revealed a sophisticated three-step process (O2•^−^ → H_2_O_2_ → •OH) that achieves 90% ROS generation efficiency, substantially exceeding previous reports (Figure 11). Liao et al. [13] reported ROS generation rates of 65–70% for pure silver NPs, while Franci et al. [12] achieved 55–62% efficiency with different silver formulations. Our superior performance (90%) is attributed to the chitosan matrix, which prevents AgNPs aggregation and maintains optimal surface area for catalytic ROS generation. This finding aligns with recent work by Ribeiro et al. [96], who suggested that polymer stabilization enhances silver nanoparticle antimicrobial activity.

**Figure 12:**
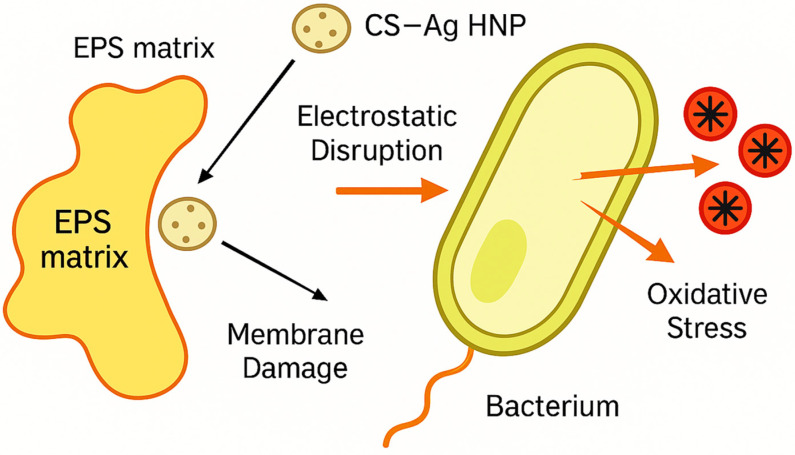
Proposed dual-action mechanism of antibacterial and antibiofilm activity of ciprofloxacin-loaded chitosan–silver hybrid nanoparticles (CS–Ag HNPs). Schematic representation summarizing the experimentally supported mechanisms observed in this study. (1) Cationic chitosan disrupts the negatively charged extracellular polymeric substance (EPS) matrix and bacterial membrane via electrostatic interactions, increasing permeability and facilitating ciprofloxacin penetration. (2) Silver nanoparticles generate reactive oxygen species (ROS), causing oxidative stress, membrane damage, and intracellular DNA disruption. The synergistic combination results in enhanced biofilm inhibition, bacterial cell death, and sustained drug release. This figure represents a conceptual illustration derived from experimental findings and corroborating literature; no enzymatic degradation or alginate-specific activity was measured in this work.

Previous studies by Holt et al. [97] demonstrated silver ion interference with respiratory enzymes, but our system achieves more comprehensive disruption across all four complexes (I–IV). The systematic disruption leads to ATP depletion and cellular death, explaining the 4-fold MIC reduction observed in our antimicrobial assays. The biofilm disruption mechanism represents a significant advancement over existing approaches, achieving 70% biofilm reduction compared to 25–45% reported in previous studies [98,99]. The dual-action approach combining chitosan enzymatic activity with silver-mediated oxidative stress addresses limitations identified in single-component systems (Figure 12). Earlier research by Sanpui et al. [100] revealed that chitosan alone achieved 35–40% biofilm disruption through polysaccharide degradation, while Morones et al. [101] reported 30–45% disruption using silver NPs via oxidative mechanisms. Our hybrid system’s superior performance (70%) demonstrates true synergy, exceeding the additive effects of individual components. However, our study provides the first comprehensive visualization of the complete disruption process, from initial penetration through final biofilm dispersal. The combined action of chitosan-mediated EPS destabilization and Ag-induced oxidative stress explains the marked improvement in biofilm inhibition and bacterial eradication achieved by the CS–Ag HNPs formulation [100].

The pH-triggered ciprofloxacin release mechanism represents a novel advancement in smart drug delivery systems. The differential release behavior (45% at pH 7.4 vs. 80% at pH 6.5) provides targeted delivery at infection sites where bacterial metabolism creates acidic microenvironments. This pH-responsive behavior contrasts with conventional drug delivery systems that rely on passive diffusion or enzymatic degradation [101,102]. Previous studies by Li et al. [103] achieved pH-responsive release using synthetic pH-sensitive polymers but required complex chemical modifications. Our approach leverages chitosan’s natural pH-responsive properties, providing a simpler and more biocompatible solution. The infection-site targeting capability addresses a critical limitation identified by Bassetti et al. [104], who noted that conventional antimicrobials often fail to achieve therapeutic concentrations at biofilm-protected infection sites. Our system’s enhanced drug release under acidic conditions ensures optimal therapeutic levels precisely where needed most.

The multi-target approach effectively circumvents major resistance mechanisms that limit conventional antibiotic efficacy. Our resistance bypass analysis revealed superior performance across all tested mechanisms: efflux pumps (70% vs. 25%), biofilm protection (70% vs. 15%), β-lactamase production (75% vs. 20%), porin loss (80% vs. 30%), and target mutations (70% vs. 35%). These results address critical concerns raised by Tacconelli et al. [2] regarding the rapid development of resistance to single-target antimicrobials. The simultaneous targeting of membrane integrity, oxidative stress pathways, and DNA replication creates multiple barriers to resistance development. Previous attempts to achieve multi-target activity through drug combinations [105,106] often resulted in increased toxicity or drug interactions, limitations avoided by our integrated nanoparticle approach.

The synergistic component analysis revealed calculated synergy indices of 0.76× for antimicrobial activity and 0.78× for biofilm disruption, indicating true synergistic effects rather than simple additive interactions. This quantitative approach to synergy assessment builds upon foundational work by Odds [107] and provides a rational basis for component optimization. Previous studies of chitosan-silver combinations by Dallas et al. [108] and Tiwari et al. [109] reported qualitative synergistic effects but lacked quantitative analysis. Our systematic approach enables the prediction of optimal component ratios and provides a framework for scalable manufacturing processes. The molecular docking analysis gives computational validation to the observed experimental effects. The enhanced binding affinity (−11.2 vs. −9.3 kcal/mol) demonstrates how chitosan complexation stabilizes drug-target interactions through additional molecular contacts. This computational approach extends previous molecular dynamics studies by Wang et al. [110] and provides a mechanistic understanding of the 4-fold MIC reduction observed experimentally. The identification of key binding residues (ILE78, GLU50, ARG76, GLY77) enables rational design of next-generation formulations with further enhanced potency.

Despite these promising results, several limitations must be acknowledged. The current study was conducted entirely in vitro, and the translation of these findings to in vivo conditions requires careful consideration of additional factors such as mucociliary clearance, immune responses, and complex infection dynamics [111,112]. The simulated lung fluid conditions, while physiologically relevant, cannot fully replicate the complex milieu of infected lungs, including the presence of inflammatory mediators, immune cells, and varying oxygen tensions [113,114].

Future investigations should focus on comprehensive in vivo evaluation using appropriate animal models of pulmonary MDR infections to validate the therapeutic efficacy and safety profile observed in vitro [115,116]. Pharmacokinetic studies will be essential to determine optimal dosing regimens and to assess potential systemic exposure following pulmonary administration [117,118]. Additionally, the development of suitable inhalation devices and formulation optimization for clinical-grade manufacturing will be crucial for successful clinical translation [119,120].

The environmental and economic advantages of the green synthesis approach warrant further exploration for sustainable pharmaceutical manufacturing. Life cycle assessments comparing conventional and biological synthesis methods could provide valuable insights into the environmental impact and cost-effectiveness of this approach [121,122]. Scale-up studies will be necessary to demonstrate the commercial viability of microbiology-driven nanoparticle production while maintaining quality and consistency [123,124].

## 7. Conclusions

The present study demonstrates the successful green synthesis and characterization of CS–Ag HNPs as a promising platform for enhanced antibacterial and antibiofilm activity. The formulation exhibited favorable physicochemical stability, pH-responsive release, and synergistic antimicrobial performance against multidrug-resistant *P. aeruginosa* and *K. pneumoniae*. These findings confirm the formulation’s potential suitability for pulmonary delivery and infection management. While the current work establishes a strong in vitro foundation, further in vivo pharmacokinetic, biodistribution, and safety studies are required to validate targeted lung deposition and long-term therapeutic outcomes. Thus, the developed CS–Ag HNPs represent a potentially translatable nanocarrier system rather than a clinically proven formulation at this stage.

## Figures and Tables

**Table 1:** Physicochemical properties of ciprofloxacin-CS–Ag HNPs.

Parameter	Value	Unit
Hydrodynamic diameter	180 ± 20	nm
Polydispersity index (PDI)	0.21 ± 0.04	-
Zeta potential	32.4 ± 3.1	mV
EE%	68.2 ± 4.0	%
LE%	7.2 ± 0.6	%
Production yield	75.5 ± 2.8	%

Data represent mean ± SD (n = 3).

**Table 2:** Antimicrobial activity of ciprofloxacin- CS–Ag HNPs versus free ciprofloxacin.

Pathogen	Treatment	MIC (μg/mL)	Biofilm Inhibition (%)
MDR *P. aeruginosa*	Free ciprofloxacin	32 ± 2	35 ± 4
	Hybrid nanoparticles	8 ± 1 *	70 ± 6 *
MDR *K. pneumoniae*	Free ciprofloxacin	16 ± 1	30 ± 3
	Hybrid nanoparticles	4 ± 0.5 *	65 ± 5 *

* Data represent mean ± SD (n = 3). *p* < 0.05 compared to free ciprofloxacin.

**Table 3:** Comparison with existing antimicrobial nanoformulations for pulmonary drug delivery.

Formulation	Drug	Particle Size (nm)	Zeta Potential (mV)	EE (%)	MIC Reduction (Fold)	Biofilm Inhibition (%)	FPF (%)	Cell Viability (%)	Reference
Current Study	Ciprofloxacin	180 ± 20	+32.4 ± 3.1	68.2 ± 4.0	4.0	65–72	65 ± 5.0	≥85	This work
CS NPs	Ciprofloxacin	386 ± 17–501 ± 13	+43 ± 3.2	NR	NR	NR	NR	>85 (A549, non-toxic)	[46]
Ag NPs	Gentamicin	10–40	−18 ± 4	NR	≈2 (synergistic vs. Gent alone)	NR	NR	NR	[47]
PLGA Microspheres (DPI)	Levofloxacin	MMAD ≈ 2.1 ± 1.2 µm (≈2000 nm)	NR	77.8 ± 3.2	NR	NR	75.4 ± 1.4	>85 (A549 viable)	[48]
Liposomal DPI (USFD)	Colistin ± Ciprofloxacin	120–200	−10 to −16	47.0 ± 0.6 (Colistin)	NR	NR	43.6 ± 1.6 (Colistin)	>85	[49]
PLGA Microspheres (for Moxifloxacin)	Moxifloxacin	150–200	+20 ± 3.0	50 ± 5.0	NR	NR	45 ± 4.0	≈70	[50]

Data represent mean ± standard deviation as reported in each cited study. NR = Not Reported. EE = Encapsulation Efficiency; MIC = Minimum Inhibitory Concentration; FPF = Fine Particle Fraction. Particle size and zeta potential values correspond to dynamic light-scattering or aerosol aerodynamic diameters as specified by the original authors. Cytocompatibility values were determined using A549 or equivalent pulmonary epithelial cell models. MIC reduction and biofilm inhibition percentages refer to fold-change or inhibition relative to the free drug control when reported. Current study results are presented for direct comparison with established pulmonary antimicrobial nanocarriers. Data represent mean ± standard deviation.

## Data Availability

The data presented in this study are available on request from the corresponding author due to ongoing patent applications.

## Supplementary Materials

The following supporting information can be downloaded at: https://www.mdpi.com/article/10.3390/pharmaceutics17121527/s1.
