# Supplementary material for: From Bacterial Extract to Breakthrough Therapy: Pseudomonas fluorescens-Enabled Green Synthesis of pH-Responsive Chitosan–Silver Hybrid Nanoparticles for Next-Generation Pulmonary Drug Delivery Anti-MDR Treatment
Source: Pharmaceutics. 2025 Nov 27;17(12):1527. doi: 10.3390/pharmaceutics17121527 (PMC12736315; doi:10.3390/pharmaceutics17121527)
Supplement: Supplementary file 1 [file pharmaceutics-17-01527-s001.zip › pharmaceutics-3933466-supplementary.pdf]

Supplementary Table S1. Checkerboard FICI Matrices

(A) Pseudomonas aeruginosa

Ciprofloxacin Fractional MIC	CS–Ag HNPs 0.25× MIC	CS–Ag HNPs 0.5× MIC	CS–Ag HNPs 1.0× MIC
**0.25**	0.56	0.61	0.74
**0.50**	0.50	0.52	0.68
**1.00**	0.63	0.65	0.82

(B) Klebsiella pneumoniae

Ciprofloxacin Fractional MIC	CS–Ag HNPs 0.25× MIC	CS–Ag HNPs 0.5× MIC	CS–Ag HNPs 1.0× MIC
**0.25**	0.60	0.62	0.76
**0.50**	0.56	0.58	0.66
**1.00**	0.69	0.72	0.84

Minimum FICI values (0.50 for *P. aeruginosa*; 0.56 for *K. pneumoniae*) indicate clear to borderline synergy.

Supplementary Table S2. Time-Kill Assay at Reduced Doses (0.5× MIC + 0.5× MIC)

Time (h)	Ciprofloxacin (0.5× MIC)	CS–Ag HNPs (0.5× MIC)	Combination (0.5×+0.5×)
**0**	0.0	0.0	0.0
**2**	–0.8	–1.0	–1.8
**4**	–1.1	–1.4	–2.6
**6**	–1.5	–1.7	–3.6
**24**	–2.3	–2.4	–4.1

≥2 $\log_{10}$ CFU/mL reduction at 6 h for the combination vs. single agents confirms synergistic bactericidal effect.

Supplementary Table S3. Comparative physicochemical and antimicrobial characteristics of ciprofloxacin-loaded chitosan nanoparticles (CS NPs, Ag-free) and chitosan–silver hybrid nanoparticles (CS–Ag HNPs).

Parameter	CS NPs (Ag-free)	CS–Ag HNPs (Hybrid)	Remarks
**Hydrodynamic diameter (nm)**	220 ± 35	180 ± 20	Ag inclusion reduced size due to ionic crosslinking and denser matrix.
**Polydispersity index (PDI)**	0.25 ± 0.05	0.21 ± 0.04	Both formulations show narrow, monomodal distributions.
**Zeta potential (mV)**	+28.5 ± 2.8	+32.4 ± 3.1	Slightly higher charge in Ag hybrid enhances stability and bacterial adhesion.
**Encapsulation efficiency (EE%)**	45.2 ± 3.5	68.2 ± 4.0	Silver–polymer network improved drug entrapment.
**Loading efficiency (LE%)**	5.1 ± 0.4	7.2 ± 0.6	Consistent with higher encapsulation efficiency.
**Production yield (%)**	72.8 ± 3.0	75.5 ± 2.8	Comparable manufacturing yield.
**MIC against MDR *P. aeruginosa* (µg/mL)**	16 ± 1	8 ± 1	2-fold improvement for hybrid system.

**MIC against MDR *K. pneumoniae* (µg/mL)**	8 ± 0.5	4 ± 0.5	2-fold improvement for hybrid system.
**Biofilm inhibition (%) – *P. aeruginosa***	42 ± 4	70 ± 6	Enhanced penetration due to Ag-induced ROS and charge effects.
**Biofilm inhibition (%) – *K. pneumoniae***	38 ± 3	65 ± 5	Confirmed synergistic antibacterial action.
**Fine particle fraction (FPF, %)**	60 ± 5	65 ± 5	Both within inhalable range (1–5 µm MMAD).
**Cell viability (Calu-3, %)**	92 ± 4	85 ± 5	Ag inclusion causes mild reduction but remains biocompatible.
**TEER retention (% of baseline)**	95 ± 3	90 ± 4	Both formulations preserve epithelial barrier integrity.

Values are expressed as mean ± SD (n = 3). CS–Ag HNPs exhibited higher encapsulation efficiency, enhanced antimicrobial and anti-biofilm performance, and suitable aerodynamic and biocompatibility characteristics. The Ag-free formulation demonstrated moderate activity, confirming the synergistic role of silver incorporation within the chitosan matrix.

Supplementary Table S4: Long-Term Stability of Lyophilized CS–Ag HNPs

Parameter	Initial (0 mo)	3 mo (25 °C / 60 % RH)	6 mo (25 °C / 60 % RH)	6 mo (40 °C / 75 % RH)
**Particle size (nm)**	182 ± 6	185 ± 8	187 ± 9	191 ± 10
**PDI**	0.25 ± 0.02	0.26 ± 0.03	0.27 ± 0.03	0.29 ± 0.03
**ζ-Potential (mV)**	+31.5 ± 1.7	+30.6 ± 2.0	+29.8 ± 2.2	+28.9 ± 2.5
**EE (%)**	68.2 ± 3.5	66.7 ± 3.8	65.9 ± 3.7	63.8 ± 4.1
**Drug content (%)**	97.8 ± 1.4	96.3 ± 1.6	95.6 ± 1.8	94.1 ± 2.0
**Visual appearance**	White, free-flowing	Unchanged	Unchanged	Slightly denser, no caking
